# Polymeric Composites with Embedded Nanocrystalline Cellulose for the Removal of Iron(II) from Contaminated Water

**DOI:** 10.3390/polym10121377

**Published:** 2018-12-12

**Authors:** Anayet Kabir, Matthew J. Dunlop, Bishnu Acharya, Rabin Bissessur, Marya Ahmed

**Affiliations:** 1 Department of Chemistry, University of Prince Edward Island, Charlottetown, PE C1A 4P3, Canada; akabir@upei.ca (A.K.); mdunlop@upei.ca (M.J.D.); rabissessur@upei.ca (R.B.); 2 Faculty of Sustainable Design & Engineering, University of Prince Edward Island, Charlottetown, PE C1A 4P3, Canada; bacharya@upei.ca

**Keywords:** polymeric composites, antifouling, metal binding, iron chelation, polydopamine coating, free-radical polymerization

## Abstract

The exponential increase in heavy metal usage for industrial applications has led to the limited supply of clean water for human needs. Iron is one of the examples of heavy metals, which is responsible for an unpleasant taste of water and its discoloration, and is also associated with elevated health risks if it persists in drinking water for a prolonged period of time. The adsorption of a soluble form of iron (Fe^2+^) from water resources is generally accomplished in the presence of natural or synthetic polymers or nanoparticles, followed by their filtration from treated water. The self-assembly of these colloidal carriers into macroarchitectures can help in achieving the facile removal of metal-chelated materials from treated water and hence can reduce the cost and improve the efficiency of the water purification process. In this study, we aim to develop a facile one-pot strategy for the synthesis of polymeric composites with embedded nanocrystalline cellulose (NCC) for the chelation of iron(II) from contaminated water. The synthesis of the polymeric composites with embedded nanoparticles was achieved by the facile coating of ionic monomers on the surface of NCC, followed by their polymerization, crosslinking, and self-assembly in the form of three-dimensional architectures at room temperature. The composites prepared were analyzed for their physiochemical properties, antifouling properties, and for their iron(II)-chelation efficacies in vitro. The results indicate that the embedded-NCC polymeric composites have antifouling properties and exhibit superior iron(II)-chelation properties at both acidic and basic conditions.

## 1. Introduction

The exponential increase in heavy metal usage due to rapid urbanization and industrialization has caused detrimental effects on human health and the environment [1,2,3,4]. According to a recent estimate, the toxicity of heavy metal ions and their contamination in water resources leads to an estimated two million casualties per year. The presence of heavy metal ions such as cadmium, mercury, chromium, lead, and iron in drinking water is associated with organ damage, birth defects, and carcinogenesis [1]. Iron is one of the most common heavy metals in the Earth’s crust, which is required for normal physiological functions of the human body, in trace amounts. However, the presence of high concentrations of iron in ground water due to natural resources or human activity and the continuous exposure of high iron content to humans is associated with elevated health risks, including organ damage, heart diseases, and ophthalmic disorders [1,2,3,4,5,6,7]. The soluble form of iron (Fe^2+^) is associated with an unpleasant taste, discoloration, turbidity, and microbial growth in water resources, and its removal from drinking water is the most cumbersome task [1,2,3,4,5,6,7]. Recently, tremendous efforts have been focused on the removal of iron(II) from water resources and some of the most studied techniques for this purpose are oxidation, electrolysis, ion exchange, adsorption, and electrocoagulation [1,2,3,4,5,6,7,8,9]. 

In comparison to the other methods, adsorption is the most economical, facile, and commonly used method for the removal of heavy metal ions from drinking water, and polymeric materials, specifically polyelectrolytes, comprise the major group of chemical adsorbents, being capable of excellent metal-chelating efficacies, due to the presence of diverse functional groups on their surface and their facile regeneration in acidic or basic conditions [2,3,4,7,9,10,11]. The incorporation of polyelectrolytes on nanoparticles further improves the metal-chelation efficacies, as the embellishment of functional group-laden polymers on the three-dimensional surface of nanomaterials enhances the metal ion-binding capacities of the resultant nanocomposites [12,13]. Owing to their biocompatibility, low toxicity, and low density, carbon-based nanoadsorbents such as carbon nanotubes, nanocrystalline cellulose (NCC), carbon aerogels, and their polymeric nanocomposites have received significant interest for water purification applications [12,13,14]. For example, the synthesis of polydopamine-coated NCC was recently reported, and these polymer-doped nanomaterials were found to exhibit superior iron(II) and iron(III)-binding efficacies [13]. However, the usage of polyelectrolytes or their nanocomposites for the chelation of heavy metal ions requires extra steps of separation, such as centrifugation, filtration, or sedimentation, to remove the metal-adsorbed colloidal carriers from purified water, which further increases the complexity and cost of the water purification system. To simplify the process of water purification, the self-assembly of nanomaterials into three-dimensional macroscopic architectures may produce diverse composites with superior properties compared to those of individual nanomaterials [12,15]. 

The self-assembly of polyelectrolytes into three-dimensional architectures has recently been reported by Kulig et al. [15], but the incorporation of nanoparticles such as NCC into these macroscopic architectures can be a complex task. The covalent attachment of polyelectrolytes on the surface of NCC, followed by their self-assembly into discrete materials, may require complex chemical reactions, which will reduce the number of active sites available on the polymer backbone, hence reducing the metal-chelation properties and self-assembly capabilities of the resultant composites. Zhi and coworkers reported the synthesis of graphene foams with lead ion-binding efficacies; these composites were prepared by electrostatic interactions between cationic moieties, tetraethylenepentamine (TEPA), and anionic carboxyl groups of graphene oxide, but the reaction was performed at high pressure and temperature [14]. 

The recent interest in the strongly adhesive properties of polydopamine coatings, along with their capability to polymerize ionic and nonionic monomers on a variety of surfaces under slightly basic conditions, may provide an alternative and facile method to coat ionic species on the surface of NCC, hence eliminating the need for complex chemical methods to achieve the surface functionalization of NCC [16,17]. Moreover, in comparison to natural polyelectrolyte-based complexes, which offer limited control regarding tuning the physiochemical properties (cation-to-anion ratio and molecular weights) of self-assembled materials, the coating of ionic monomers on the surface of NCC, followed by their facile polymerization, crosslinking, and self-assembly may provide greater control of the physiochemical properties of resultant composites, and this may translate into their superior iron(II)-chelation properties. In this study, we aim to develop polymeric composites with embedded NCC by a one-pot method, by coating NCC with polydopamine under slightly basic conditions. Polydopamine-coated NCC was then decorated with ionic monomers, namely methacrylic acid and tetraethylenepentamine methacrylamide (TEPMA), followed by their self-assembly, polymerization, and crosslinking in the presence of *N,N′-*methylenebisacrylamide by a free-radical polymerization method to yield polymeric composites with embedded NCC which have optimal strength. The composites produced were characterized by Fourier transform infrared spectroscopy (FTIR), thermogravimetric analysis (TGA), scanning electron microscopy (SEM), and X-ray diffraction analysis (XRD) and were studied for iron(II)-chelation efficacies and antifouling properties in vitro. To the best of our knowledge, this is the first report describing the preparation of polyelectrolytes with embedded nanoparticles by the physical and chemical crosslinking of ionic monomers on the surface of nanoparticles, in which the materials are studied for their iron(II)-chelation efficacies in situ.

## 2. Experimental Procedure

### 2.1. Materials

Nanocrystalline cellulose was extracted from tunicates according to a previously established procedure [18]. Briefly, club tunicates were collected from Malpeque Bay, Prince Edward Island (PEI). The tunicate samples were taken from several mussel socks, immediately placed into freezer bags, and frozen. Frozen samples were thawed in a fume hood. The tunic was then liberated from the internal organs of the tunicate using a scalpel. The tunic samples were extensively washed with deionized water. Crystalline cellulose was then isolated from club and vase tunicates isolated from Malpeque Bay, PEI, via the prehydrolysis–kraft cooking–bleaching method. The prehydrolysis, kraft cooking, and bleaching steps were done in a PARR 4748 Large Capacity Acid Digestion Bomb equipped with polytetrafluoroethylene (PTFE) liner. Prehydrolysis and kraft cooking reactions were each carried out at 180 °C for 2 h. The bleaching step was carried out at 75 °C for a 1-h duration. A 20:1 ratio of solid to solution was used. The reactor was heated to temperature in a Paragon Sentry Xpress 4.0 oven, and agitation was accomplished by physical shaking of the reactor at 10-minute intervals. Products were isolated via vacuum filtration using medium-pore-size fritted filters. The product was then dried in a vacuum oven at 60 °C for 24 h. The isolation and drying method was kept consistent for each step. The crystalline cellulose obtained following the bleaching step was centrifuged at 10,000 rpm and dialyzed against deionized water for 5 days to purify the product. Ultrasonication of the purified crystalline cellulose resulted in the formation of NCC [18]. The length of the NCC used was 1567 ± 638 nm and the diameter was 18.3 ± 10.1 nm.

Tetraethylenepentamine (TEPA), methacrylic anhydride, hydroquinone (HQ), methacrylic acid (MA), potassium persulfate (KPS), *N,N,N′,N′-*tetramethylethylenediamine (TEMEDA), and *N,N′-*methylenebisacrylamide were purchased from Sigma-Aldrich (Canada). Dopamine hydrochloride (DOPA) was purchased from Alfa Aesar, (Canada). Methanol, acetone, 2-propanol, and ammonium hydroxide (NH_4_OH) were purchased from Fisher Scientific (Canada).

### 2.2. Synthesis of Tetraethylenepentamine Methacrylamide (TEPMA·2HCl)

In brief, 5 mmol of TEPA was dissolved in 2 mL of methanol in the presence of small amount (~1 mg) of hydroquinone, followed by the addition of 6.5 mmol of methacrylic anhydride dropwise into the solution. The solution was stirred overnight. Next day, the solution was protonated with 2.5 mL of 12.1 M hydrochloric acid (HCl) and methanol was removed under reduced pressure. The creamy substance was crystallized with acetone and the dried solid obtained was stirred in the presence of 10 mL of 2-propanol in a 70 °C preheated oil bath for 20 min to remove multi-methacrylamide amines. The crude TEPMA obtained was dissolved in 10 mL of methanol at room temperature for 20 min and the mixture was filtered to obtain pure TEPMA·2HCl at 70% yield. The purified TEPMA salt was analyzed by ^1^H-NMR and ^13^C NMR using a Bruker 300 MHz NMR instrument. The molar mass of the monomer was obtained by electron spray ionization mass spectrophotometry (ESI-MS) (University of Toronto mass spectrophotometer facility).

The ESI-MS result reveals that the molecular weight of the pure TEPMA salt is 326.6 g/mol, which corresponds to TEPMA·2HCl formation.

^1^H NMR (D_2_O, ppm): δ 1.8 (s, 3H, CH_3_), 3.2–3.8 (m, 16H, CH_2_), 5.6 (s, 1H, C=CH_2_), 5.8 (s, 1H, C=CH_2_).

DEPT-45 spectrum of ^13^C-NMR (D_2_O, ppm): δ 17.8, 35.6, 36.4, 43.4, 43.6, 43.7, 43.9, 44.9, 48.5, 122.4.

### 2.3. Synthesis of Composites

NCC stock solution was prepared at 10% *w*/*v* in deionized water. The commercially available methacrylic acid was passed through a silica column to remove any inhibitor. Then, 1 mL of NCC stock solution was coated with 68 µmol of DOPA in the presence of 1.14 mM NH_4_OH and the solution was stirred for 20 min. The reaction was cooled to 0 °C and varying concentrations of TEPMA·2HCl and methacrylic acid were added in the presence of 10–20 mol % *N,N′-*methylenebisacrylamide (cross-linker), 10 μM potassium persulfate, and 20 µL TEMEDA. The formation of composites was confirmed by vial inversion test and the gelation time was recorded. The stoichiometric details of the synthesis of NCC-embedded polymeric composites are summarized in Table 1. The composites prepared were washed three times with deionized water and were vacuum-dried at room temperature to obtain pure materials. Sample 5 (polymeric composite 5 (PC5) in Table 1) resulted in the successful formation of the desired composites and was further subjected to thermal, mechanical, and physical characterization and was evaluated for its iron-chelation efficacies in situ.

Polydopamine-coated NCC was prepared as a control, by reacting 10 mg/mL of NCC with 68 µmol of DOPA in the presence of 1.14 mM NH_4_OH and stirring the solution for 20 min. Polydopamine (PDA)-coated NCC was purified by centrifugation and was washed with deionized water three times to remove any unreacted material. PDA-coated NCC was dried and used for further characterization and analysis.

### 2.4. Protein Absorption Capacity of Composites

The protein absorption capacity of the NCC-embedded polymeric composites was measured as follows: Bovine serum albumin (BSA) (500 μg/mL) was incubated with 4 mg/mL of composites in phosphate buffer saline (PBS) buffer (pH 7.4) for 24 h. The composites were then washed 3 times, the supernatant was discarded, and the composites were immersed in PBS for 24 h to release any imbibed protein. The supernatant was then analyzed for the presence of BSA using the bicinchoninic acid (BCA) assay, according to the manufacturer’s protocol.

### 2.5. Evaluation of Iron Ion-Binding Capacity

Ferrous sulfate was used as the iron source for the metal-binding experiments, and the amount of the Fe^2+^ ion adsorbed by the composites was measured by UV spectrophotometry, according to a recently reported protocol [19]. The general procedure for the iron-binding (Fe^2+^) analysis is given below.

Ferrous sulfate solutions of various concentrations (1.37–30 μg/mL) were prepared in a 0.23 mM sodium chloride and 0.31 μM citric acid stock solution of pH 3.8 and 10, respectively. The mixture was incubated for three hours and the pH of the resultant suspension was adjusted to 3.8. Then, 0.3% (*w*/*w*) aqueous phenanthroline and 10.0% (*w*/*w*) aqueous hydroxylamine hydrochloride was added into the solution. The solution was stirred for 5 minutes and was allowed to stand for another 3 h. The absorption spectrum of the solution was recorded at 508 nm, and a calibration curve of the absorbance versus concentration of iron(II) was prepared (see Supporting Information, Figure S6). To evaluate the efficacies of polymeric composites for iron(II)-chelation, 4 mg/mL concentration of dried NCC composites were added into the ferrous sulfate stock solutions with different pH (3.8 and 10) and the mixtures were incubated for various time periods (1 min–72 h). The pH of the filtrate was adjusted to 3.8 and the amount of iron in the supernatant was evaluated as described above. The equilibrium adsorption of the composites (Qe, mg/g) was calculated by taking into consideration the initial amount of iron in the solution and the amount of iron present in supernatant after the adsorption, according to Equation (1); the metal removal efficiency % (R%) was calculated by Equation (2); and the adsorption capacity (Q_t_) at time ‘t’ was calculated by Equation (3):

Q_e_ = [(C_0_ − C_e_) × V]/m (1)

R (%) = [C_0_ − C/m] × 100 (2)

Q_t_ = [(C_0_ − C_t_) × V]/m (3)

where C_0_ is the initial concentration, C_t_ is the concentration at given time t, and C_e_ is the equilibrium concentration of metal ions (mg/L) in solution; V is the total volume of solution (L); and m is the mass of composites [14,20,21]. 

### 2.6. Sorption Kinetics

To describe the sorption kinetics, pseudo-first-order kinetic and pseudo-second-order kinetic models were tested. The linear form of pseudo-first-order kinetics was used for fitting the sorption kinetics and is formulated below:

ln (q_e_ − q_t_) = lnq_e_ − k_1_t (4)

where q_e_ and q_t_ are the amount of metal ions absorbed in mg/g at equilibrium and at time ‘t’, respectively. k_1_ is the rate constant of the sorption per hour. The graph of ln (q_e_ − q_t_) versus t was plotted, and k_1_ and ln q_e_ were calculated from the slope and intercept of the line, respectively.

The pseudo-second-order kinetic model is expressed as:

t/q_t_ = 1/k_2_ (q_e_)^2^ + (1/q_e_) t (5)

The graph of t/q_t_ versus t was plotted, and q_e_ (theoretical value) and k_2_ (rate constant in g/mg/hr) was calculated from the slope and intercept of the line, respectively.

Sorption isotherms were fitted by linear form of the Langmuir isotherm:

C_e_/Q_e_ = C_e_/q_m_ + 1/K_L_ q_m_ (6)

where q_m_ is the maximum absorption capacity of polymeric composites embedded with NCCs (mg/g), C_e_ is the equilibrium concentration of the adsorbate in solution (mg/L), and K_L_ is the Langmuir affinity constant (L/mg).

The data were also fitted by the linear form of the Freundlich model:

ln q_e_ = ln K_F_+ 1/*n* ln C_e_ (7)

where K_F_ is the Freundlich constant of the relative adsorption capacity of the adsorbent and 1/*n* indicates the heterogeneity factor or adsorption intensity [20,21].

### 2.7. Characterization Techniques

#### 2.7.1. Powder X-ray Diffraction (XRD)

XRD diffractograms of the NCC composite samples were recorded using a Bruker AXS D8 Advance instrument, which was equipped with a graphite monochromator, variable divergence slit, variable antiscatter slit, and a scintillation detector. Cu (Kα) radiation (λ = 1.524 Å) was used, and the measurements were performed in air at room temperature from 2°–60° (2θ). Dried samples were milled to powder and deposited on double-sided scotch tape, which was mounted on glass slides.

#### 2.7.2. Thermogravimetric Analysis (TGA)

The thermal properties of composites were studied using TGA (TA Instruments TGA Q500). The samples were heated from 25 to 650 °C at a heating rate of 10 °C/min under an atmosphere of 40 mL/min nitrogen and 60 mL/min air.

#### 2.7.3. Scanning Electron Microscopy (SEM)

To assess the surface morphology via scanning electron microscopy (SEM), micrographs were obtained on a Delong America LVEM5 Benchtop SEM instrument. Powdered samples were deposited onto stubs and held in place with carbon tape. All samples were sputter-coated with 60:40 Au/Pd prior to imaging using a SPI-Module Sputter Coater (SPI Supplies).

#### 2.7.4. Attenuated Total Reflection Fourier Transform Infrared Spectroscopy (ATR-FTIR)

ATR-FTIR was performed on a Bruker Alpha-T single-reflection attenuated ATR module equipped with a platinum–diamond crystal. Samples were dried, milled to powder, and immediately analyzed. Spectra were collected from 16 scans per sample and subtracted from a background spectra between 375–4000 cm^−1^ at a resolution of 0.9 cm^−1^.

## 3. Results and Discussion

### 3.1. Synthesis of NCC-Embedded Composites

**Scheme 1:**
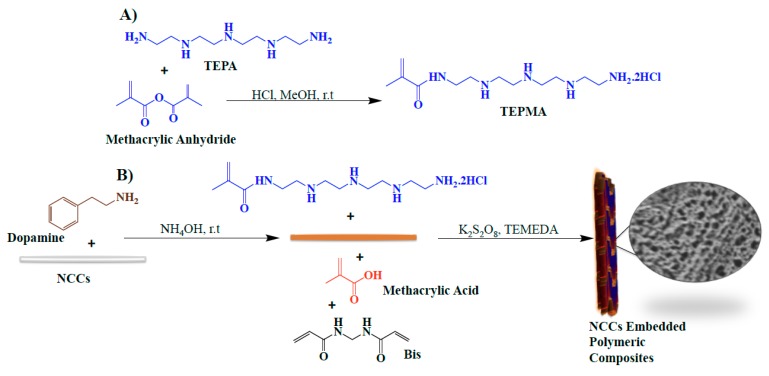
Schematics depicting (**A**) the synthesis of TEPMA·2HCl and (**B**) the synthesis of polymeric composites with embedded nanocrystalline cellulose (NCC) via the polydopamine coating of NCC, followed by the incorporation of ionic monomers, their polymerization, crosslinking, and self-assembly at room temperature (r.t): Tetraethylenepentamine (TEPA), Tetraethylenepentamine Methacrylamide (TEPMA·2HCl), *N,N,N′,N′-*tetramethylethylenediamine (TEMEDA).

Polymer composites with embedded nanoparticles been the subject of much interest for the development of novel materials with improved mechanical and physiochemical properties [22,23,24,25,26]. The synthesis of polymeric composites with embedded NCC generally involves the mixing of nanoparticles in preformed polymeric solution, followed by their chemical crosslinking to yield the desired composites. However, the packing of NCC into polymeric materials often provides limited advantages, due to the problems in achieving high concentrations of NCC in polymeric solutions and issues associated with the compatibility of functional groups on the surface of NCC with those of the polymeric materials [22,23,24,25]. In an effort to develop NCC–polyelectrolyte-complexed composites, we utilized the strongly adhesive properties of polydopamine to anchor ionic monomers on the surface of NCC, followed by their polymerization, crosslinking, and self-assembly at room temperature (Scheme 1). The first step in the synthesis of polyelectrolyte composites with embedded nanoparticles for the study of iron(II)-chelation efficacies is the choice of ionic monomers. The research on water-purifying materials indicates that amine-containing moieties, namely tetraethylenepentamine (TEPA) and carboxylic acid-based monomers, such as methacrylic acid, are among the most studied ionic materials for water purification applications [14,26,27,28,29]. The incorporation of TEPA into nanocomposites is generally obtained by electrostatic interactions between amine groups of TEPA and anionic groups of composites (carboxyl, sulfate), followed by their crosslinking at high temperature and pressure to obtain macroarchitectures [14]. We argue that the conversion of TEPA into a methacrylamide-based monomer, namely tetraethylenepentamine methacrylamide (TEPMA), may present a simple and convenient method to develop amine-functionalized polymeric composites by the free-radical polymerization method. The synthesis of TEPMA was achieved by the modification of TEPA in the presence of methacrylic anhydride, followed by the precipitation of the TEPMA salt (TEPMA·2HCl) in methanol. The synthesis of the TEPMA salt is depicted in Scheme 1. The chemical structure of pure TEPMA·2HCl was confirmed by FTIR, ^1^H NMR, and ^13^C NMR. ESI-MS provides the molar mass of the TEPMA salt and indicates the degree of protonation of TEPMA as TEPMA·2HCl (Supporting Information, Figures S1–S4).

The synthesized ionic monomers are then functionalized on the surface of NCC by utilizing the strongly adhesive properties of dopamine. NCC are coated with polydopamine, followed by their attachment with ionic monomers (TEPMA·2HCl and methacrylic acid), polymerization, and crosslinking in a one-pot reaction in the presence of *N,N’-*methylenebisacrylamide (Scheme 1 and Supporting Information, Figure S5). Polymeric composites with embedded NCC were allowed to self-assemble in the form of macrocomposites, and the gelation of composites was confirmed by vial inversion test and gelation time was recorded (Table 1). The colloidal nature of the polydopamine-coated NCC composites and the well-defined three-dimensional architecture of the NCC–polymer composites are compared in photographs (Supporting Information, Figure S6).

The molar ratios of the monomers (TEPMA, methacrylic acid) and crosslinker were optimized to obtain polymeric composites of optimal strength (Table 1). As shown in Table 1, a critical molar ratio of 1:1 of amine/carboxyl groups (that is, a 2:1 molar ratio of methacrylic acid/TEPMA·2HCl) is required to achieve the polymeric composites in the form of thixotropic gels, highlighting the role of electrostatic interactions in the structure of composites (Table 1). Further increases in both the crosslinking density (20 mol %) and the molar ratio of methacrylic acid in the composites led to significant reduction in the gelling time and exhibited improved strength of the resultant materials, suggesting that both chemical crosslinking and ionic interactions are critically important to obtain embedded-nanoparticle polymeric composites of high strength. It should be noted that the coating of NCC with one type of ionic monomer, namely methacrylic acid or TEPMA, did not produce polymeric composites of optimal strength at any of the studied concentrations, possibly due to the electrostatic repulsion of polymer chains formed, which impaired the self-assembly or setting behavior of composites. This further highlights the role of electrostatic interactions in the assembly of composites achieved (Supporting Information, Figure S7). The composite with reduced gelling time and of optimum strength (the sample indicated as PC5 (polymeric composite 5) in Table 1) was chosen for physiochemical characterization and for the study of iron(II)-binding efficacies in vitro. The polymeric composites prepared in the absence of NCC showed poor gelation properties and significantly low yield (~20%), indicating that the concentration of ionic monomers used for composite formation was too low to form an ionic gel and that NCC provides the major scaffold for the coating of ionic monomers and for facilitating their polymerization into polymeric composites. 

### 3.2. Characterization of Composites

**Figure 1:**
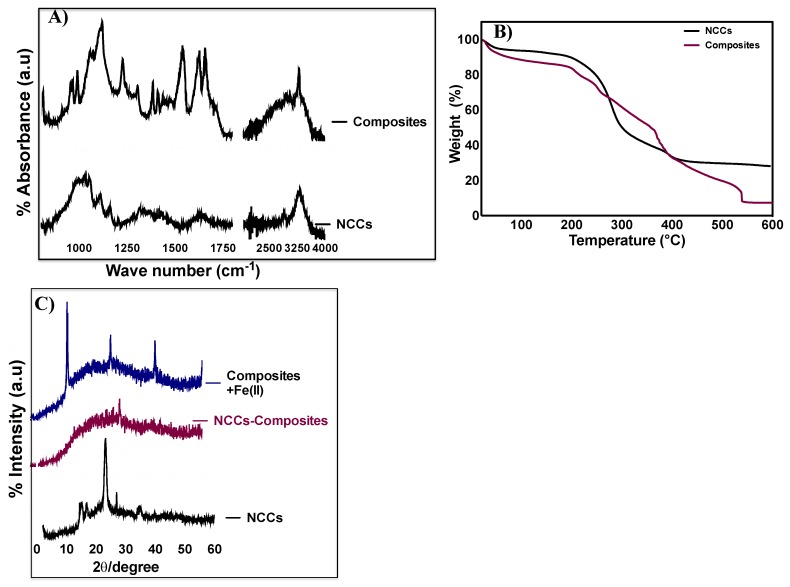
(**A**) FTIR spectra of NCC and embedded-NCC polymeric composite. (**B**) TGA analysis of NCC and embedded-NCC composite. (**C**) XRD patterns of NCC, embedded-NCC polymeric composite, and iron-loaded composite.

The polymeric composite (sample PC5 in Table 1) was characterized regarding its surface morphology by FTIR spectroscopy and XRD analysis (Figure 1A,C). The FTIR spectra of NCC reveal two sharp peaks at 1100 and 3250 cm^−1^, indicating the stretching of the C–O and O–H groups in the NCC structure [26,27]. The subsequent polymerization and crosslinking of TEPMA·2HCl and methacrylic acid on the surface of NCC in the presence of polydopamine results in polymeric composites with the appearance of functional groups associated with ionic monomers. The broadening of the 3250 cm^−1^ peak indicates the presence of bound O–H groups of methacrylic acid. The presence of peaks at 1388 and 1420 cm^−1^ are due to the symmetric and asymmetric bending vibrations of NH_2_, and the strong band at 1658 cm^−1^ is due to the bending vibrations of N(R)H in the TEPMA structure [14,26]. The peak at 1130 cm^−1^ indicates the C–O stretching of methacrylic acid, and the asymmetric stretching of C=O is indicated by the vibrational peak at 1725 cm^−1^ [26,27] (Figure 1A). The crystalline structure of the materials was then analyzed by XRD. 

**Figure 2:**
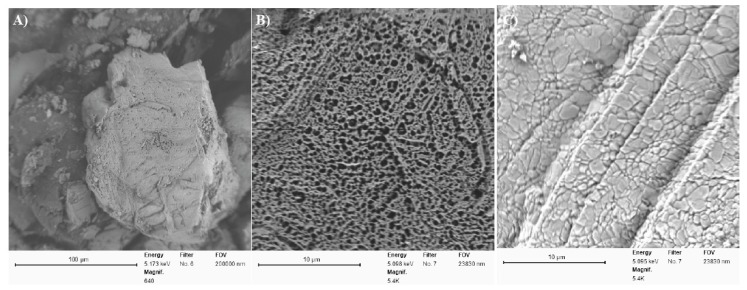
SEM images of polymeric composite (**A**) at low magnification (100 μm) and (**B**) high magnification (10 μm), and (**C**) high-magnification (10 μm) SEM images of iron-loaded polymeric composite.

XRD patterns of NCC revealed a typical cellulose-I structure, as indicated by 2θ = 16.5°, 22.4°, and 34.6° [27] (Figure 1C). In comparison to the longitudinal fiber-like, crystalline morphology of NCC, the embedded-NCC polymeric composite prepared at the NCC concentration of 10 mg/mL exhibited a completely amorphous structure, which may indicate that concentration of NCC in the composite was significantly low and the diffraction of cellulose nanocrystals was not observed. (Figure 1C and Supporting Information, Figure S8). The modification of NCC with polymeric structures is well known to amorphize or mask the crystalline structure of NCC [24]. We also report that the embedded-NCC composite yielded a clay-like amorphous structure. The surface morphology of the embedded-NCC composite as studied by SEM further confirms the amorphous clay-like nature of the composite (Figure 2). SEM images of the composite obtained at higher magnification unveiled a highly porous structure, which is considered ideal for the heavy metal adsorption behavior of polymeric composites (Figure 2A,B). The presence of metal-chelating groups (hydroxyl, amine, and carboxyl groups) and the highly porous structure of embedded-NCC polymeric composites warrant the study of their metal ion-binding efficacies [1,14,25,26,27,30]. The composite prepared was incubated with 60 mg/L of iron(II) for the period of three hours, and iron-loaded composite was evaluated for its physiochemical properties by XRD and SEM. Figure 1C reveals characteristic differences in the XRD patterns of the iron-loaded and iron-free composite. The appearance of crystalline peaks in the XRD patterns of iron(II)-loaded composite may be associated with the presence of iron(II) on the surface of the composite (Supporting Information, Figure S9). Furthermore, the smooth, nonporous surface of the iron-loaded composite as observed by SEM may suggest the packing of iron(II) into the multifunctional porous surface of the polymeric composite (Figure 2C). 

The thermal properties of the composite were then studied by TGA. As expected, the embedded-NCC composite showed higher thermal stability in comparison to NCC alone (Figure 1B). TGA analysis revealed the higher stability of NCC at lower temperatures (less than 200 °C); however, at temperatures above 200 °C, rapid weight loss (more than 60%) was observed due to the thermal degradation of NCC in the oxidizing atmosphere. The embedded-NCC composite, in contrast, exhibited a slower and characteristic three-step degradation profile. At low temperatures (less than 200 °C), the slight changes in the weight of the composite (~20%) were due to the loss of moisture entrapped in the composite’s architecture. The slow and continuous weight loss from 200 to 400 °C (~40%) indicates the disruption of ionic interactions within the composite. The complete thermal degradation of the composite occurred from 400–520 °C, and can be attributed to the breakage of covalent bonds in the composites at high temperatures [22].

### 3.3. pH-Dependent Iron-Binding Capabilities of the Composite

**Figure 3:**
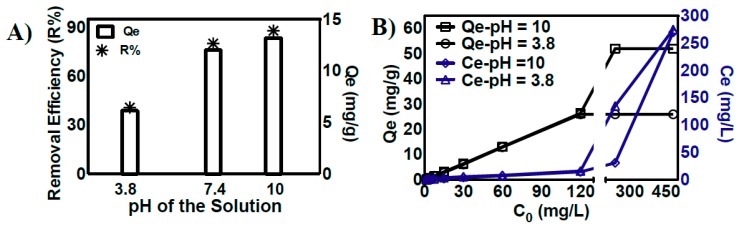
(**A**) Effect of pH on the amount of iron adsorbed at equilibrium (Qe, mg/g) and its removal efficiency (R%) in the presence of a constant amount (4 g/L) of polymeric composite. (**B**) Analysis of iron adsorbed at equilibrium (Qe, mg/g) on the surface of the composite and the concentration of free iron (Ce), as a function of iron concentration in solution, at pH 3.8 and pH 10.

The binding of the soluble form of iron (Fe^2+^) on the surface of the composite was investigated in detail as a function of the initial concentration of iron in solution, at both acidic and basic pH (Figure 3A and Supporting Information, Figure S10). The pH of the solution is one of the most important factors, affecting the biosorption of iron(II) ions onto the surface of composites [7,20]. Zhang and coworkers indicated that an increase in the pH of the solution increased the amount of iron adsorbed onto rice husk materials, possibly due to the electrostatic interactions of the materials with cationic metal ions [20]. Moreover, at high pH values, the conversion of iron(II) into its hydroxide forms may lead to the precipitation and adsorption of iron(II) on the surface of composites, hence facilitating its removal [20]. In contrast, Yan et al. reported that decreased iron(II) adsorption on the surface of magnetic graphene oxide at highly acidic pH is due to the electrostatic repulsion between protonated anionic groups and metal ions. Similarly, strongly basic pH resulted in the competitive binding of hydroxyl and metal ions in solution, hence reducing the metal-chelation properties of materials [7]. We evaluated the role of solution pH in the removal of iron(II) in the presence of a constant amount of composite (4 g/L). As expected, the increase in the pH of the solution increased the iron(II)-binding efficacies of the composite. At acidic pH, the embedded-NCC composite showed low iron-chelating efficacies (R% = 30%–32%). However, at high pH, the iron-binding efficacy of the composite was increased by ~3-fold and the equilibrium iron-binding capacity of the composite increased from 30% to 89% (Figure 3A). This increase in the iron(II)-removal efficacies of the composite at basic pH was attributed to the activation (deprotonation) of carboxyl and amine groups of the composite, hence exhibiting superior iron(II)-binding capabilities by electrostatic interactions [7,31]. At acidic pH, in contrast, metal-chelating groups of the composite are in protonated form and electrostatic repulsion between the cationic metal ions and functional groups reduces their metal ion-binding efficacies. 

### 3.4. Sorption Isotherms of the Embedded-NCC Composite

The adsorption isotherms were then evaluated from the relationship between the amount of metal ions adsorbed on the surface of materials and that of the solution at equilibrium. These isotherms play a fundamental role in describing the nature of interactions between the sorbent and metal ions [7,14,20,21]. The adsorption isotherms of the composite for the removal of iron at acidic and basic pH were evaluated at a wide range of initial metal ion concentrations (Figure 3B). The increase in the concentration of iron(II) from 1.75–120 mg/L at pH 3.8 linearly increases the amount of iron adsorbed on the surface of the composite from 1–32.6 mg/g, and low concentrations of soluble iron (less than 10 mg/L) were observed in the solution form. However, further increase in the initial iron concentration of the solution (240–720 mg/L) led to the complete saturation of active metal-binding sites on the surface of the composite, and the adsorption of iron(II) achieved equilibrium. The iron-binding capacities of magnetic graphene oxide showed similar adsorption behavior, and complete saturation of metal-binding sites was observed at the saturation capacity of 43 mg/g of nanocomposite [7]. The iron(II)-binding efficacies of the embedded-NCC polymeric composite at basic pH yielded a similar trend; however, an increase in iron-binding capacity (Qe of 52.5 mg/g and 90% removal efficiency) was observed due to the stronger electrostatic interactions between the iron ions and deprotonated functional groups of the polymeric composites [21]. The iron-binding capacity of the composite at equilibrium was found to be 32.3 mg/g and 52.5 mg/g at pH = 3.8 and pH = 10, respectively (Figure 3B). The iron-binding analysis at equilibrium was subjected to the Langmuir and Freundlich sorption isotherms to investigate the adsorption mechanism of the composite (Table 2 and Supporting Information, Figure S11).

The fitting results for the adsorption of iron (II) on the surface of the composite are summarized in Table 2. The correlation coefficients of linear form of Langmuir isotherm showed poor agreement with the experimental data. However, the Freundlich isotherm was found to be suitable (R^2^ = 0.96 at pH = 3.8 and R^2^ = 0.99 at pH = 10) to explain the mechanism of iron(II) adsorption on the surface of the composite. The Freundlich isotherm is one of the most widely used mathematical descriptions, which gives an expression of surface heterogeneity and indicates the presence of active sites and their energies on the surface of materials [7]. The conformity of the data with the Freundlich isotherm suggests the multisite sorption capacity of the embedded-NCC polymeric composite, possibly due to the presence of various functional groups (hydroxyl, amine, carboxyl) on the surface of the composite with undoubtedly different metal ion adsorption capacities. This may present a heterogenous surface of the composite that follows the Freundlich isotherm [7]. Moreover, the stronger interactions between the polymeric composite and metal ions calculated from the Freundlich isotherm (n = 1.1; n >1 indicates stronger affinity) suggests that the sorption of iron(II) is facilitated on the surface of the composite [21] (Table 2). PEG-blended chitosan surfaces prepared for the chelation of iron(II) from contaminated water resources also showed strong correlation with the Freundlich isotherm and revealed that the adsorption of iron on the chitosan membranes was a heterogeneous process [28,29,31,32].

In summary, sorption isotherms analysis suggests that the Freundlich mechanism is the predominant method of iron(II) adsorption on the surface of the polymeric composite, and although pH has no significant affect on the mechanism of adsorption, the adsorption of iron on the surface of the composite is indeed facilitated at high pH. This effect is possibly attributed to the adsorption of iron(II) on the surface of the composite by electrostatic interactions between carboxyl groups of methacrylic acid [23]. The maximum iron-binding capacity of the composite was 26.5 mg/g at pH 3.8 and was 52.5 mg/g at pH 10. The maximum iron adsorption capacity of the polymeric composite was found to be similar to or even higher than some of the recently reported materials evaluated for their iron adsorption capabilities (Table 3).

### 3.5. Adsorption Kinetics of the Composite

**Figure 4:**
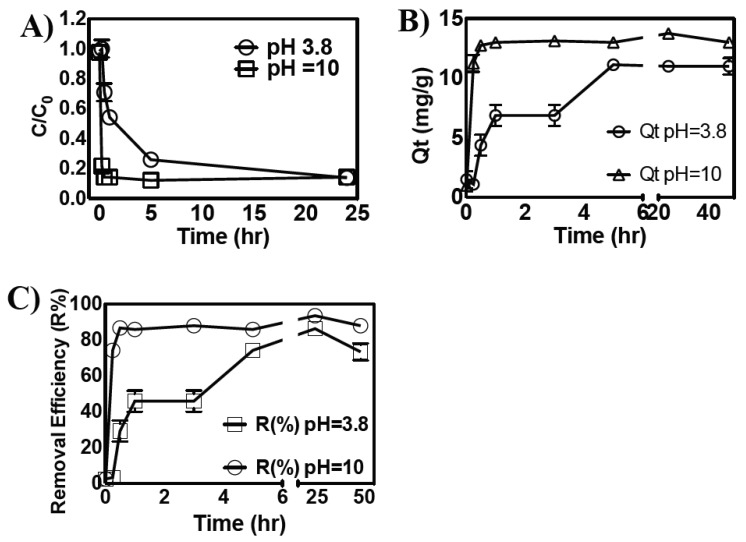
(**A**) Change in the initial concentration of iron(II) as a function of time in the presence of composite at pH 3.8 and 10; (**B**) effect of contact time on the iron adsorption capacity of the composite at pH 3.8 and 10; and (**C**) iron removal efficiency of the composite as a function of time at pH 3.8 and 10.

The change in the initial concentration of iron salt in the presence of the polymeric composite and the adsorption of iron(II) on the surface of the composite was studied as a function of time at both acidic and basic pH (3.8 and 10) (Figure 4A,B). The results indicate that the adsorption of iron on the surface of the composite was significantly faster at basic pH and that ~80% of iron was adsorbed on the composite’s surface within 15 min of incubation. The adsorption of iron at acidic pH (3.8), however, was relatively slow, and maximum adsorption was achieved in a 24-h time period. The kinetics of iron adsorption was then evaluated using pseudo-first-order and pseudo-second-order kinetic models using a constant amount of composite (4 g/L) and 60 mg/L of iron solution at 25 °C. For the applicability of the pseudo-first-order kinetic model, ln(q_e_ − q_t_) versus t should give a straight line; however, the fitting of iron adsorption data for the polymeric composite showed poor agreement with pseudo-first-order kinetics (R^2^ = 0.18 at pH = 3.8 and R^2^ = 0.46 at pH = 10), hence the pseudo-first-order kinetic model was deemed unsuitable to describe the study (Table 4). For the analysis with pseudo-second-order kinetics, the plot of t/q_t_ versus t should provide a straight line [32]. As shown in Table 3 (and Supporting Information, Figure S12), the iron adsorption data fits well with pseudo-second-order kinetics and the calculated q_ecalc_. = 11.68 mg/g is in close agreement with the experimentally obtained q_e_ (13.6 mg/g) at any studied pH.

### 3.6. Reusability and Antifouling Properties of the Composite

**Figure 5:**
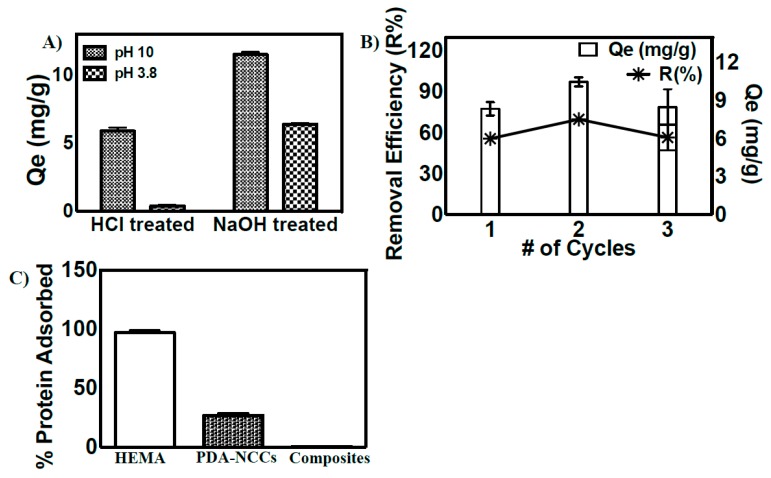
(**A**) The evaluation of the reusability of the composite at acidic and basic pH; (**B**) Iron absorption (shown by Qe) and desorption (shown by R%) on the surface of the polymeric composite after multiple cycles of NaOH treatment; (**C**) Evaluation of % protein adsorption efficacies on the surface of hydrogels (HEMA gels), polydopamine-coated NCC, and on the polymeric composite.

The reusability of metal-chelating composites is of utmost importance for their economical and practical applications [20,21]. Since the iron(II) uptake of composites is strongly pH-dependent, we evaluated the role of acidic and basic pH on the reusability of the material. The composite was first saturated with iron(II), then treated with dilute acid or base, followed by a subsequent assessment of its iron-binding capacities. The iron-binding capacities of the recycled composite showed a significant difference in the iron adsorption capabilities of the composite, depending upon the method of treatment used to remove the adsorbed iron from the composite. As shown in Figure 5, acid-treated composite showed poor iron-binding efficacies at acidic pH, while in contrast, iron(II)-binding efficacies of base-treated composite were unchanged or even higher at basic pH (Figure 5A). We suggest that improved iron(II)-binding efficacies at basic pH are due to the facile removal of adsorbed iron, along with the deprotonation of multiple functional groups present on the surface of composites, which maintained superior iron-chelation properties of the materials upon repeated exposure to iron-containing solutions (Figure 5B).

The antifouling behavior of metal-chelating materials is another important parameter to consider to evaluate their potential for water purification applications. The adsorption of organic matter on the surface of composites indeed reduces their metal ion-binding capacities and hence their applicability in water resources for prolonged periods of time [1,2,3,4,5,6,7]. We evaluated the adsorption of bovine serum albumin (BSA) (one of the most abundant proteins, which is well known for its high absorption behavior) on the surface of the polymeric composite. The adsorption behavior of BSA on the surface of the composite was compared with the adsorption of BSA on the surface of polydopamine-coated NCC and with a 2-hydroxyethyl methacrylate (HEMA)-based hydrogel. HEMA gels were used as a positive control and are well known for their protein-absorbing capabilities [37]. As expected, HEMA gels exhibited superior protein adsorption behavior. Although slight adsorption of BSA (~17%) on the surface of polydopamine-coated NCC was observed, the embedded-NCC polymeric composite showed negligible adsorption of protein on its surface (>1%) (Figure 5C). The slight adsorption of BSA on the surface of polydopamine-coated NCC may be attributed to the strong adhesion capacity of polydopamine [16,17]. The poor interactions of BSA on the surface of the polymeric composite further highlights its applicability for the chelation of iron(II) from water resources.

## 4. Conclusions

The study provides a simple and efficient method for the synthesis of polymeric composites embedded with nanoparticles with good metal ion-binding efficacies. The amorphous polymeric composite prepared at optimized amine-to-carboxyl group molar ratios exhibited a highly porous structure and superior capability for iron(II) chelation. The mechanism of iron adsorption on the surface of the polymeric composite was explained by the Freundlich isotherm. The analysis of the kinetics of iron adsorption on the surface of the composite revealed that the iron adsorption followed pseudo-second-order kinetics, and calculated q_e_ values were in strong agreement with experimental data. The results also revealed that the pH of the solution did not alter the mechanism of adsorption and its kinetics; however, the adsorption of iron on the anionic surface of the composite was facilitated at basic pH. The superior iron-binding capacities of the polymeric composite and its excellent antifouling properties suggest that this material is suitable for water purification applications.

## Figures and Tables

**Table 1:** The stoichiometric ratios of monomers and crosslinker studied for the synthesis of polymeric composites.

Sample ID	NCC (mg/mL)	TEPMA:MA (μM)	Crosslinker (mol %)	Gelation Time (min)	Gel Morphology
PC1	10	90:90	10	N/A	N/A
PC2	10	90:180	10	45	Thixotropic gel
PC3	10	90:180	20	30	Thixotropic gel
PC4	10	90:360	10	45	Thixotropic gel
PC5	10	90:360	20	15	Hard gel

**Table 2:** Sorption isotherms of iron(II) on the surface of the composite at pH = 3.8 and 10.

Langmuir Isotherm		Freundlich Isotherm		
pH	R^2^	R^2^	n	K_F_
3.8	0.347	0.96	1.1	0.044
10	0.145	0.99	1.18	0.068

Where, n represents the heterogeneity factor and K_F_ is Freundlich Isotherm.

**Table 3:** Summary of iron-chelating adsorbents evaluated in research.

Material	pH Studied	Maximum Fe-Removal Capacity (mg/g)	Reference
Embedded-NCC polymeric composites	3.8, 10	26.5, 52.5	This work
Magnetic graphene oxide	5.5	43.3	[7]
Rice husk ash	5	6.2	[20]
Chitosan–PEG blends	5–10	90.9	[31]
Natural zeolite	7	1.1	[32]
Activated charcoal	4–7	0.8	[33]
Granular activated carbon	Not provided	3.6	[34]
Apatite	4–6.6	57.1–124.2	[35]
Bentonite clay		7.09	[36]

**Table 4:** Kinetics of iron(II) adsorption on the surface of composites.

	Pseudo-First-Order Kinetics	Pseudo-Second-Order Kinetics		
	R^2^	R^2^	K_2_ (g/mg/hr)	Q_ecalc._ (mg/g)
pH = 3.8	0.178	0.98	0.034	11.68
pH = 10	0.498	0.98	0.043	11.54

## Supplementary Materials

The following are available online at http://www.mdpi.com/2073-4360/10/12/1377/s1.
