# Supplementary material for: Polymeric Composites with Embedded Nanocrystalline Cellulose for the Removal of Iron(II) from Contaminated Water
Source: Polymers (Basel). 2018 Dec 12;10(12):1377. doi: 10.3390/polym10121377 (PMC6401701; doi:10.3390/polym10121377)
Supplement: Supplementary file 1 [file polymers-10-01377-s001.pdf]

Nanocrystalline Cellulose Embedded, Polymeric Composites for Removal of Iron(II) from Contaminated Water

Anayet Kabir[1], Matthew J Dunlop[1,2], Nauman Nazeer[2], Bishnu Acharya[2], Rabin Bissessur[1] & Marya Ahmed[1,2]*

[1]*Department of Chemistry, University of Prince Edward Island, Charlottetown, PEI, C1A 4P3*

[2]*Faculty of Sustainable Design & Engineering, University of Prince Edward Island, Charlottetown, PEI, C1A 4P3*

Corresponding author: marahmed@upei.ca

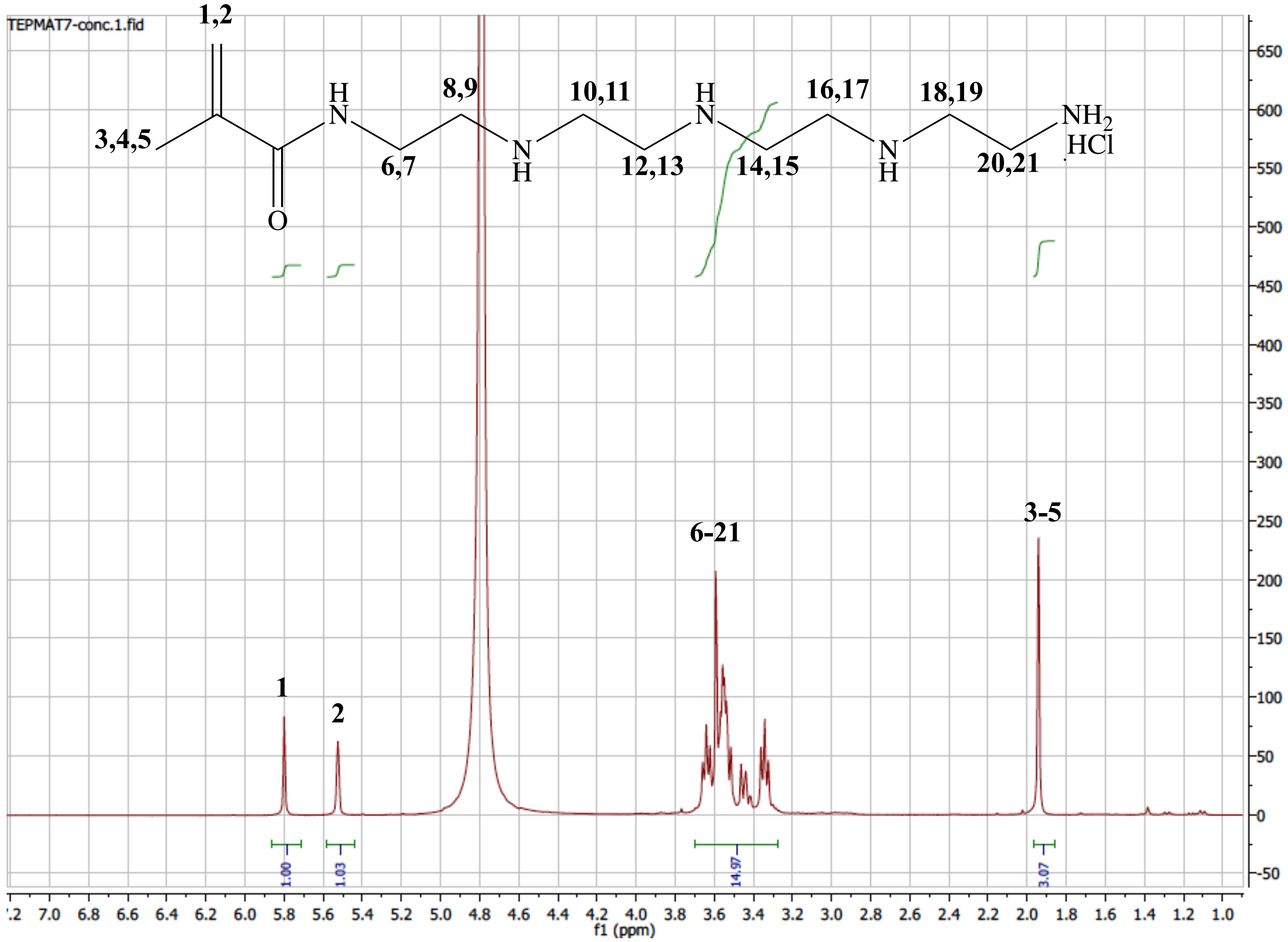

FigureS1: ^{1}H-NMR spectrum of TEPMA.2HCl in D_2O.

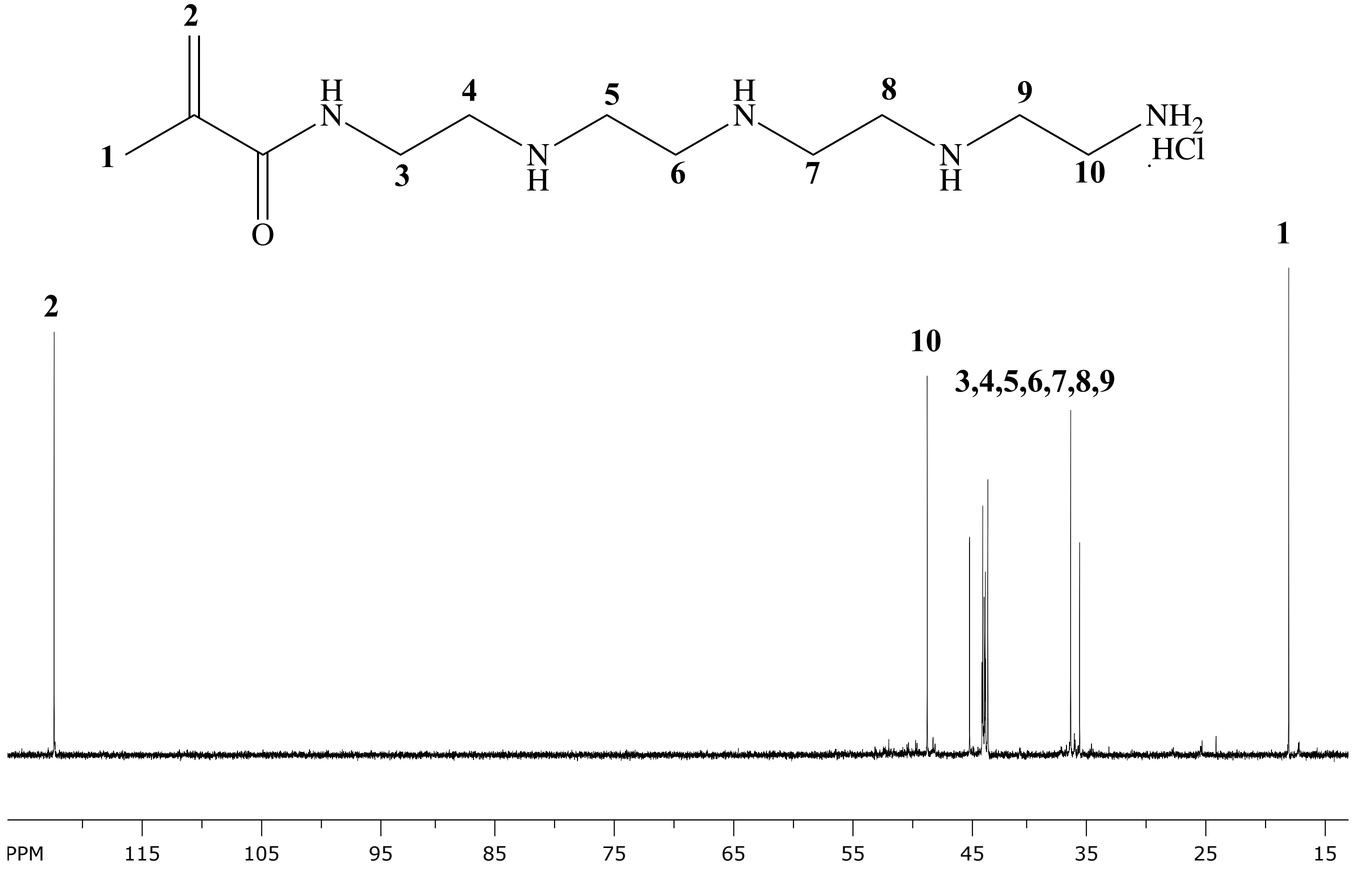

Figure S2: ^{13}C-NMR spectra of TEPMA.2HCl in D_2O.

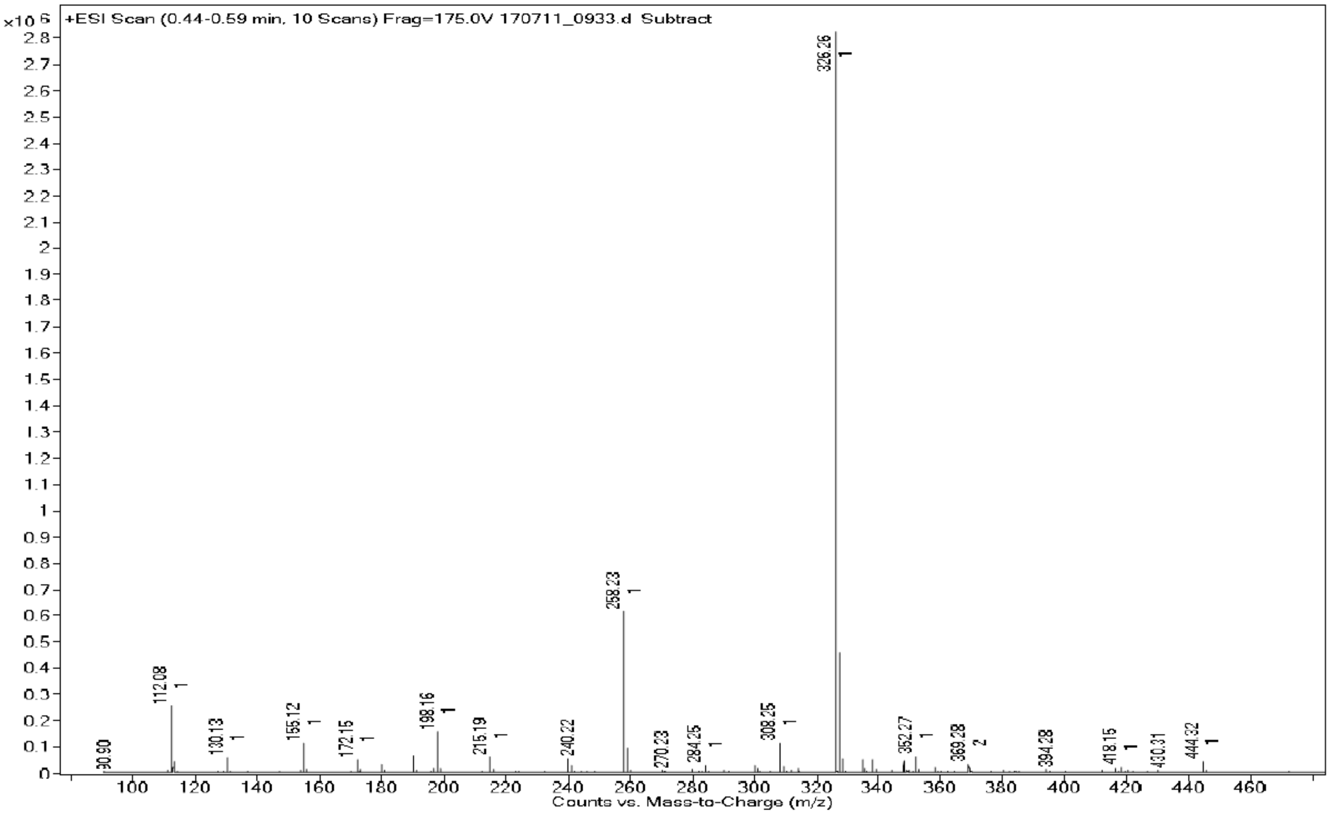

Figure S3: ESI-MS of TEPMA. 2HCl.

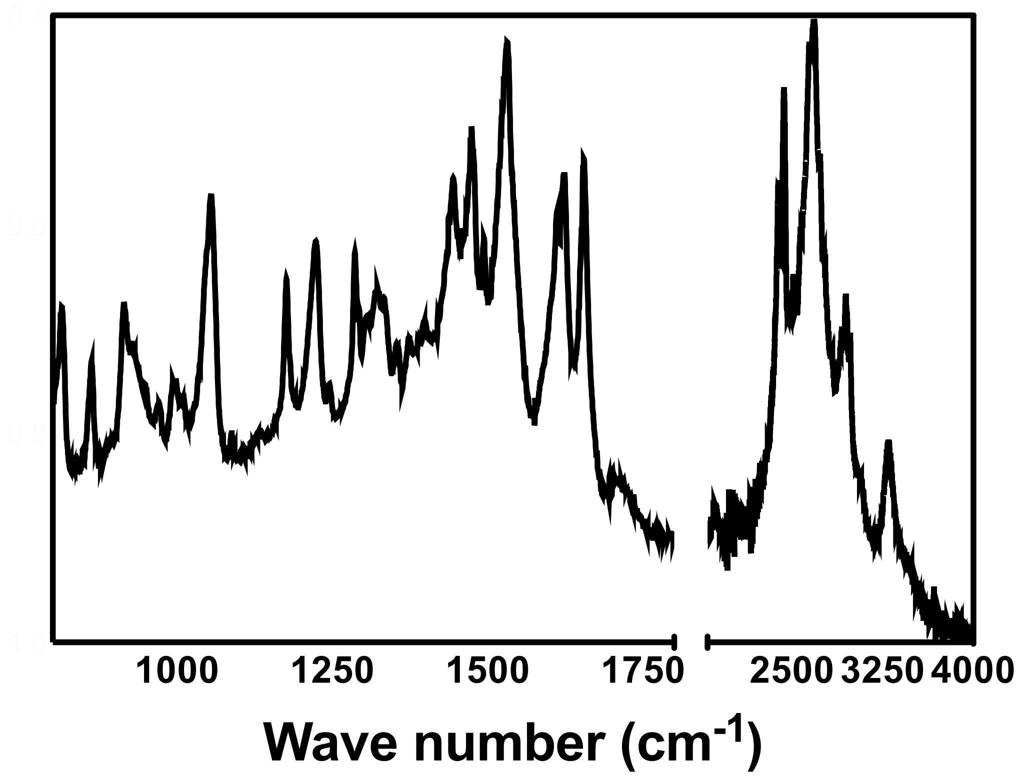

Figure S4: FTIR spectra of TEPMA.2HCl.

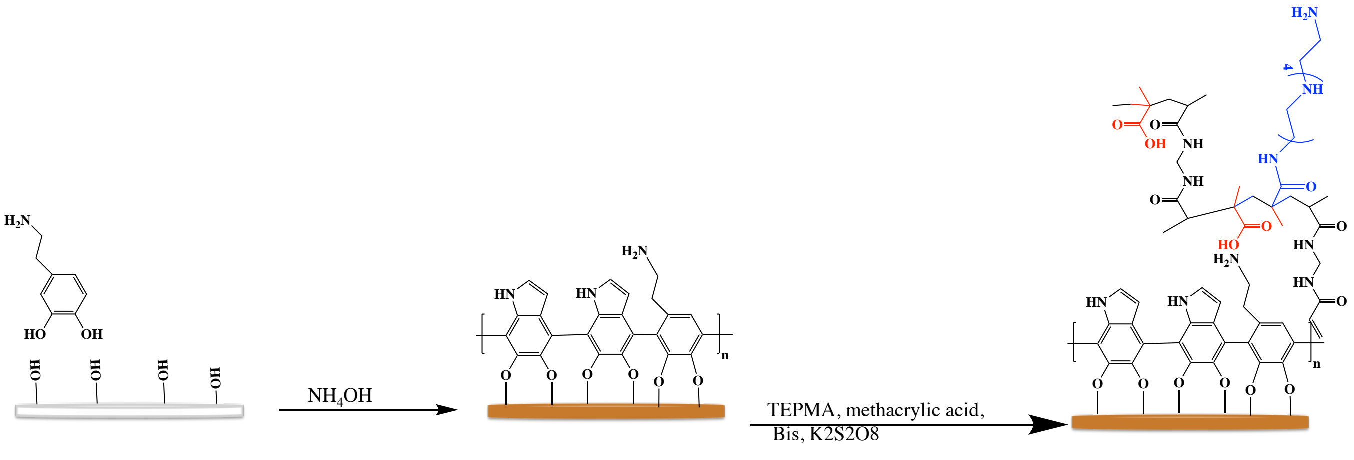

Figure S5: Simplified schematics depicting the polymerization of dopamine on the surface of NCCs, the interactions of DOPA-Coated NCCs with ionic monomers, and their crosslinking in the form of composites.

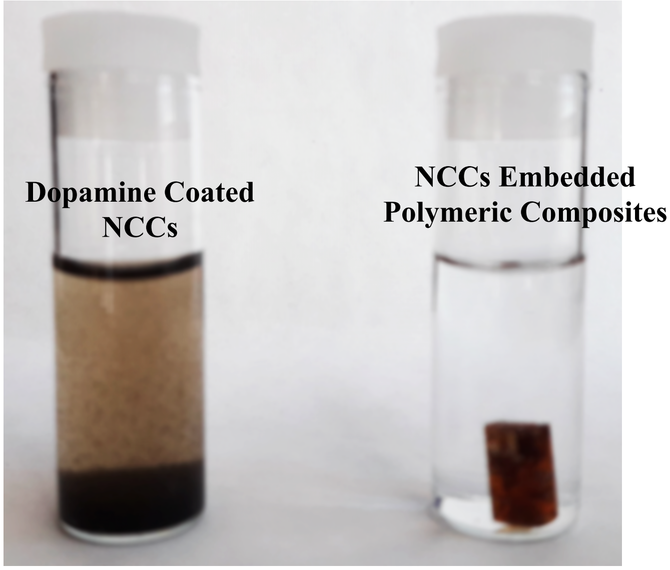

Figure S6: Photograph of polydopamine coated NCCs and NCCs embedded polymeric composites (PC5) in deionized water.

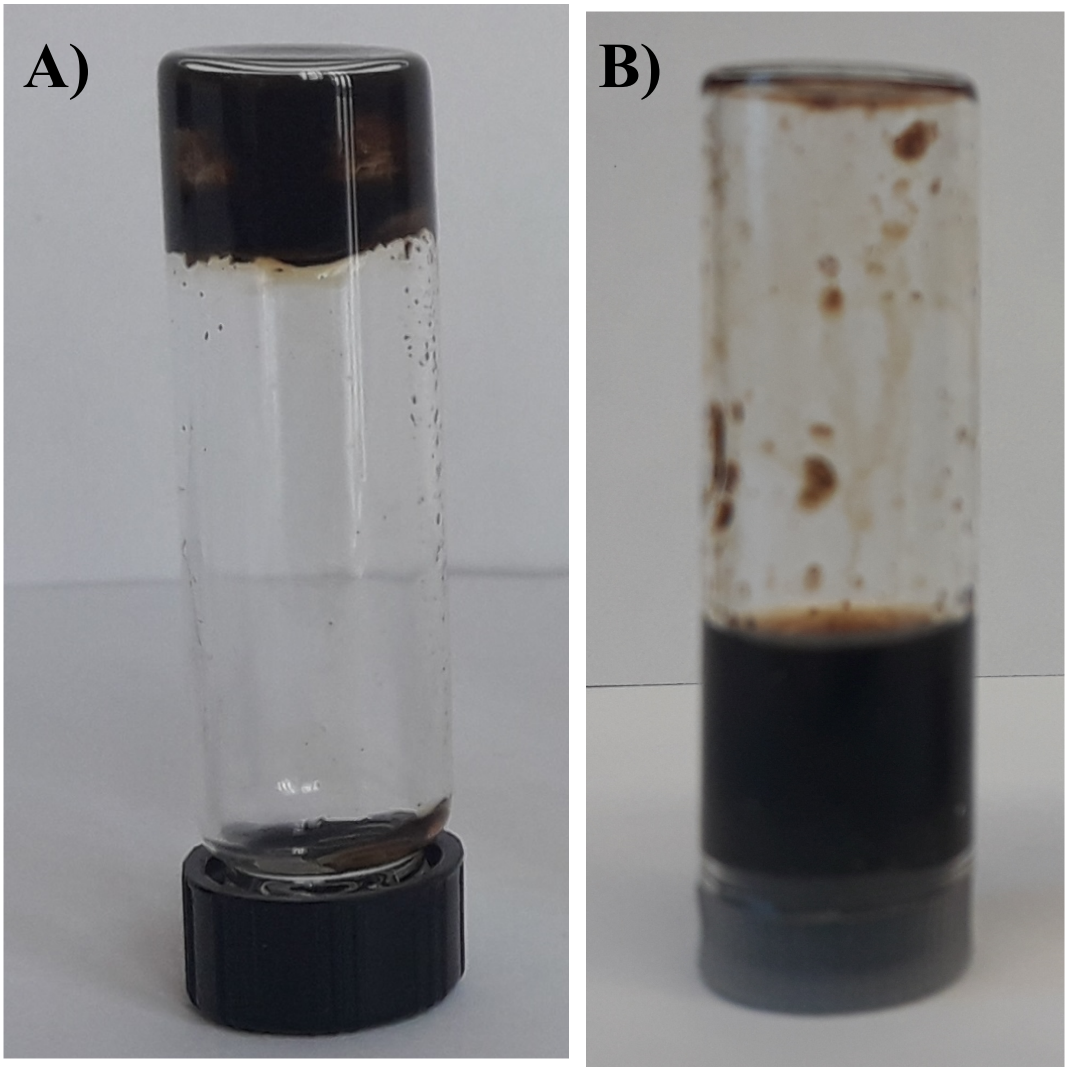

Figure S7: Vial inversion testing confirming the synthesis of NCCs embedded polymeric composites A) for sample PC5 (TEPMA:Methacrylic acid molar ratio 4:1) and B) composites prepared with 360 μM concentration of methacrylic acid coated NCCs (in the absence of TEPMA).

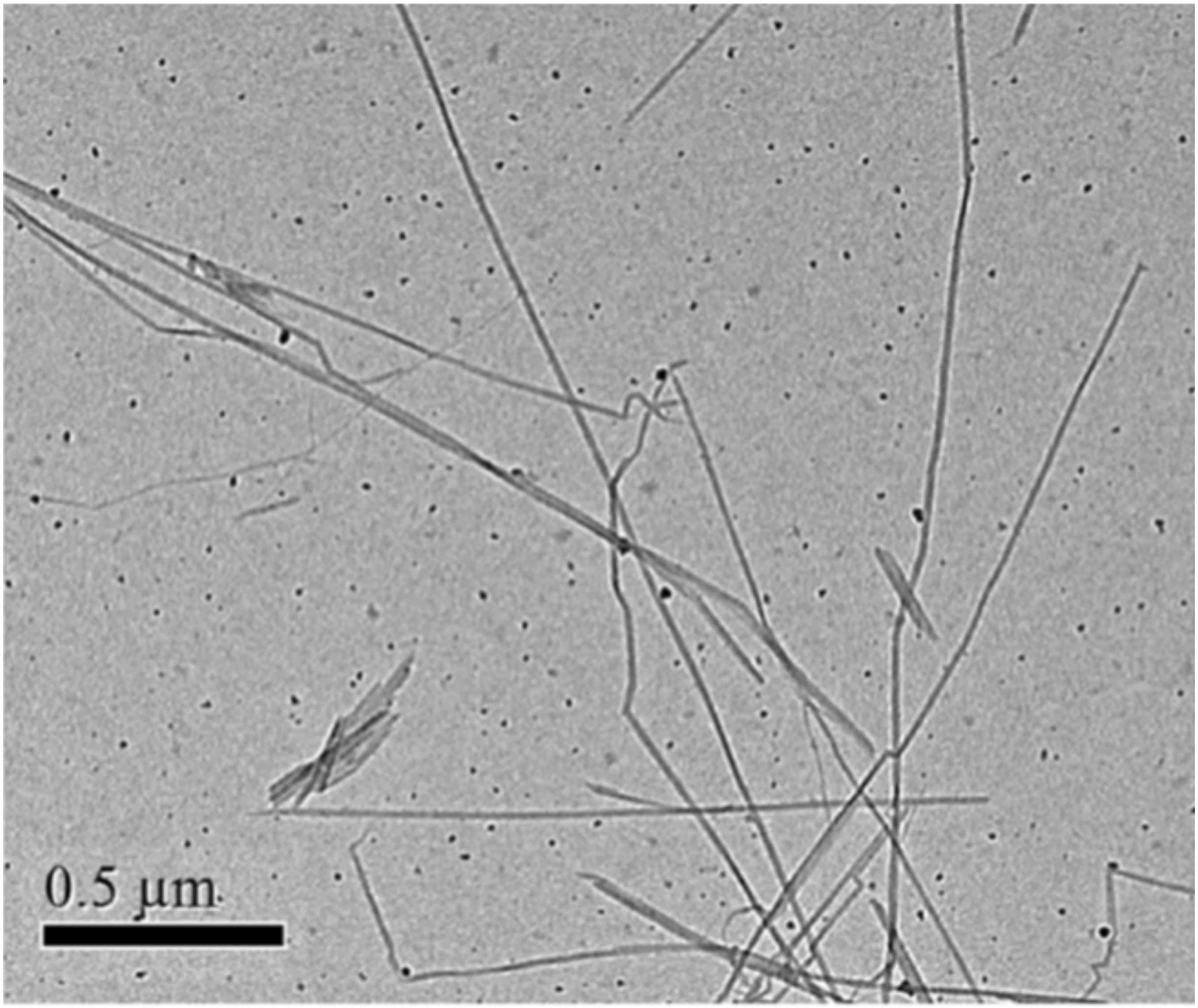

Figure S8: SEM image of NCCs extracted from tunicates.

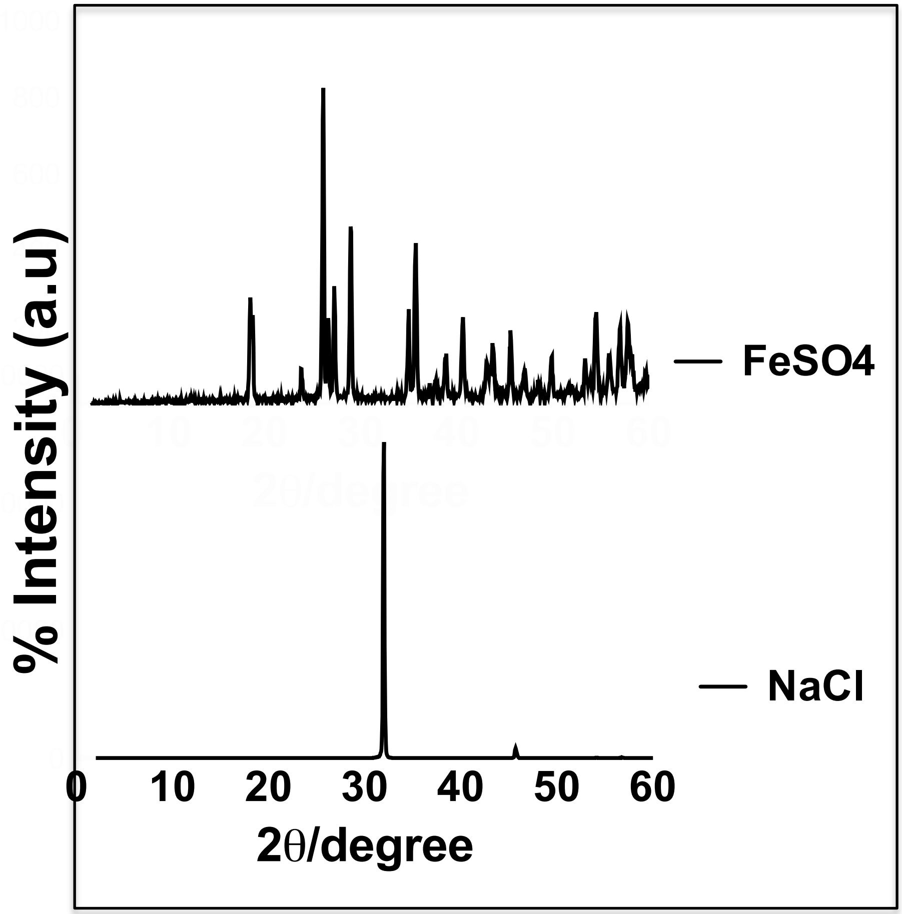

Figure S9: XRD patterns of NaCl and $FeSO_4$.

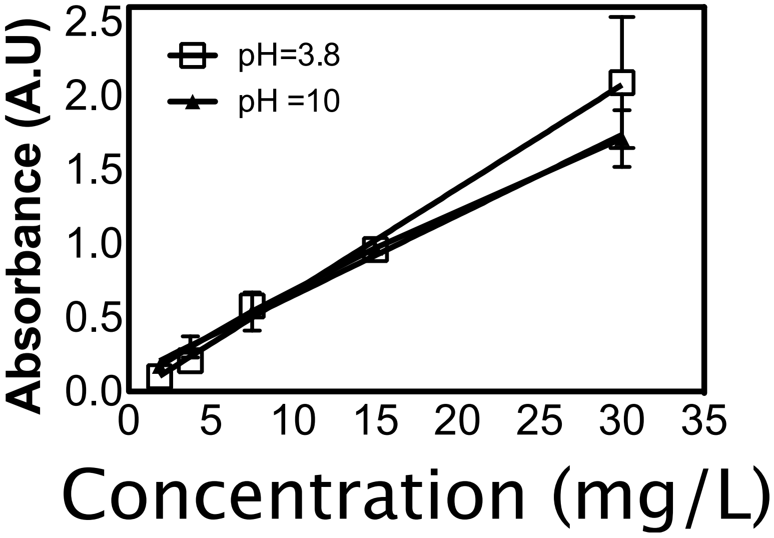

Figure S10: Calibration curve of Iron (II) as a function of concentration at pH = 3.8 & 10.

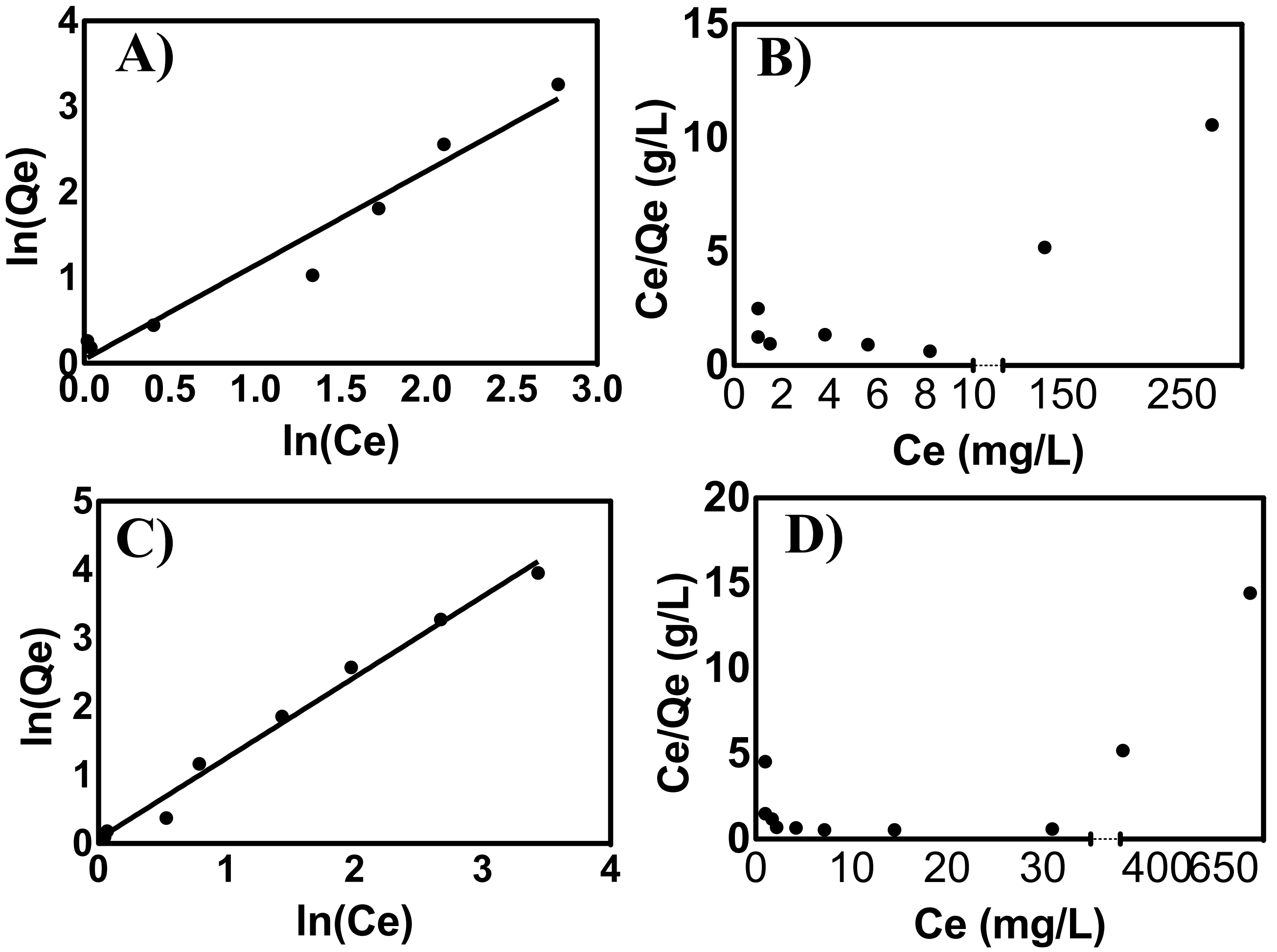

Figure S11: Adsorption isotherm plots and curve fittings by Frenudlich fitting (A) at pH 3.8 and (C) at pH 10, and by Langmuir fitting (B) at pH 3.8 and (D) at pH 10.

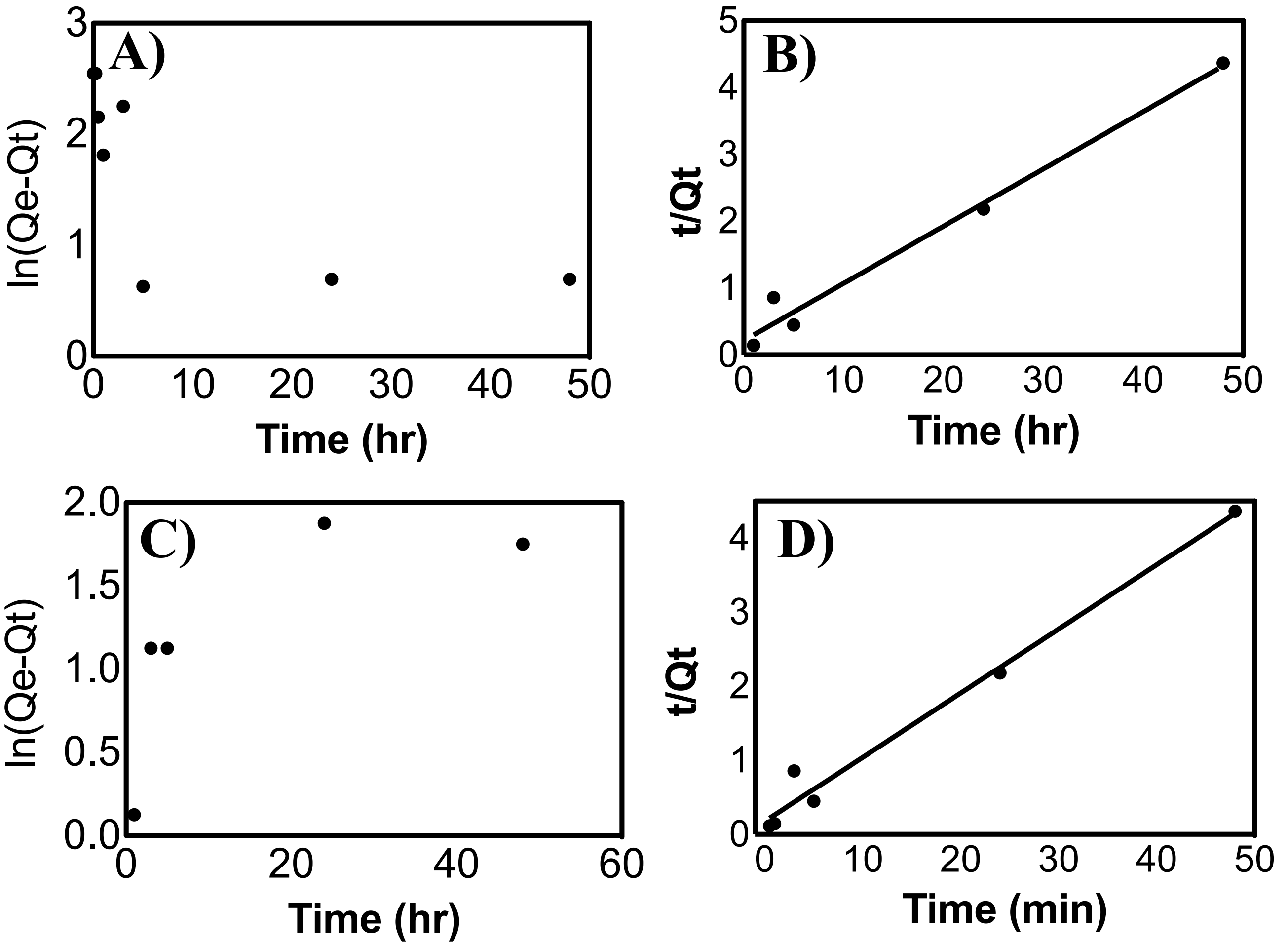

Figure S12: Adsorption kinetics plots and curve fittings for pseudo first order (A) at pH 3.8 and (C) at pH 10, for pseudo second-order kinetics (B) at pH 3.8 and (D) at pH 10.
